# A Triple-Resonance NMR Strategy for the Selective Detection of NAD^+^ and NADH Derived from a ^13^C/^15^N-Nicotinamide Riboside Probe in the Liver Extracts of Mice

**DOI:** 10.3390/s26092714

**Published:** 2026-04-28

**Authors:** Hiroki Shimada, Yusei Shinohara, Yoshihiro Uto, Hisatsugu Yamada

**Affiliations:** Graduate School of Technology, Industrial and Social Sciences, Tokushima University, 2-1 Minamijyosanjima-cho, Tokushima 770-8506, Japan; hirokishimada10.19@gmail.com (H.S.); shinohara.yuusei@tokushima-u.ac.jp (Y.S.); uto.yoshihiro@tokushima-u.ac.jp (Y.U.)

**Keywords:** triple-resonance NMR, stable isotope-enriched molecular probe, labeled nicotinamide riboside, NAD^+^/NADH

## Abstract

Alterations in the ratio of oxidized and reduced nicotinamide adenine dinucleotide (NAD^+^/NADH) reflect the intracellular redox state and have been implicated in a broad spectrum of pathological conditions, including neurogenetic disorders, heart failure, and liver diseases. In the present study, we demonstrate the selective detection of probe-derived NAD^+^ and NADH signals in mouse liver extracts by means of a triple-resonance nuclear magnetic resonance (NMR) spectroscopy technique. We prepared ^13^C/^15^N-enriched nicotinamide riboside (^13^C/^15^N-NR), which undergoes enzymatic conversion to NAD^+^ and NADH in the liver, and detected these labeled metabolites by ^1^H–{^13^C–^15^N} triple-resonance NMR measurements. This study provides a methodological proof-of-concept for the selective detection of NAD-related signals derived from a stable-isotope labeled probe in mouse liver extracts.

## 1. Introduction

As an electron carrier in living cells, nicotinamide adenine dinucleotide (NAD) is an essential cofactor for maintaining homeostasis across diverse biological processes. Interconversion between its oxidized form (NAD^+^) and reduced form (NADH) is central to NAD(H)-dependent dehydrogenase-mediated redox reactions in the mitochondria and nucleus [1]. Recently, alterations in the NAD^+^/NADH balance have been associated with intracellular redox dysregulation and various pathological conditions, including neurogenetic disorders [2], heart failure [3], and liver diseases [4]. Recent reviews have further emphasized the complexity of NAD^+^ metabolism and the importance of analytical approaches for monitoring NAD-related metabolites in biologically relevant settings [5].

Because the NAD^+^/NADH ratio is closely linked to these disease states, several fundamentally distinct approaches, including mass spectrometry (MS) [6], electrochemical sensing [6], and fluorometric detection [6,7,8,9,10], have been developed to assess this parameter. Among these, fluorometric techniques based on enzymatic cycling assays have frequently been used to estimate the intracellular NAD^+^/NADH ratio. However, these methods are limited in their capacity to analyze NAD(H) with high analytical accuracy and reproducibility, as they require enzyme-mediated redox reactions in tissue lysates and indirect estimation of individual NAD(H) levels through the detection of fluorescent products [8,9]. Accordingly, a pressing need remains for molecular monitoring techniques capable of selectively detecting NAD(H)-related signals in complex biological samples.

Nuclear magnetic resonance (NMR) spectroscopy offers considerable potential for the direct monitoring of biomolecules, providing reliable molecular identification along with high specificity. In recent years, NMR-based approaches have been used not only for structural analysis, but also for biochemical sensing and metabolic monitoring in complex biological systems [11,12,13]. In addition, isotope-assisted NMR methods have provided useful molecular information in biochemical and molecular recognition studies [14,15,16].

Multiple-resonance NMR enables the selective detection of specific protons within a ^1^H–^13^C–^15^N spin system (^1^H–{^13^C–^15^N} triple-resonance NMR) through magnetic coherence transfer [17]. This technique permits the efficient suppression of background signals arising from endogenous ^1^H-containing species in complex biological mixtures and allows for the selective detection of protons derived from ^13^C/^15^N-enriched compounds. In practice, triple-resonance NMR methodologies have been successfully applied to the monitoring of such metabolic substrates [17,18,19,20,21,22].

**Figure 1:**
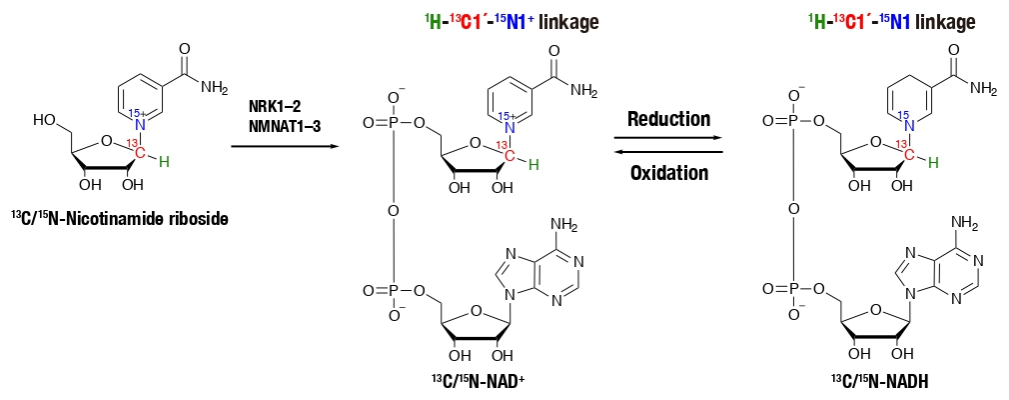
Structure of ^13^C/^15^N-doubly-labeled nicotinamide riboside (^13^C/^15^N-NR) and schematic illustration of its metabolic conversion into doubly-labeled NAD^+^ and NADH.

In the present study, we applied triple-resonance NMR to develop a probe-based strategy for the selective detection of NAD^+^- and NADH-related signals using doubly-labeled ^13^C/^15^N-nicotinamide riboside (NR), a well-established precursor of NAD^+^. As shown in Figure 1, doubly-labeled NR and its metabolites, NAD^+^ and NADH, all contain a ^1^H–^13^C–^15^N sequence, indicating that these molecules should be detectable by one-dimensional (1D) ^1^H–^13^C–^15^N triple-resonance NMR analysis. NR undergoes two sequential enzymatic transformations: phosphorylation to nicotinamide mononucleotide (NMN) via nicotinamide riboside kinase (NRK1–2) and conversion to NAD^+^ through the action of NMN adenylyltransferase (NMNAT1–3) [23]. Throughout this metabolic pathway, the ^1^H–^13^C1′–^15^N1^+^ linkage, corresponding to the site of double labeling (^13^C and ^15^N), remains intact (Figure 1). Furthermore, the reduction of the ^15^N-labeled pyridyl ring in NAD^+^ induces a pronounced upfield chemical shift of the methine proton within the ^1^H–^13^C1′–^15^N1 spin system in NADH [24]. Therefore, we hypothesized that this probe would enable selective and orthogonal detection of probe-derived NAD^+^ and NADH signals and provide a methodological proof-of-concept for monitoring NAD-related redox changes by triple-resonance NMR.

## 2. Experimental Section

### 2.1. General

Reagents and solvents were purchased from standard suppliers and used without further purification. Labeled nicotinamide (^15^N-nicotinamide) was prepared from nicotinamide in two steps using labeled ammonium chloride (^15^NH_4_Cl) (98%, ^15^N) [25]. Routine NMR spectra were obtained on a JNM-ECA500WB (500 MHz) or a JNM-ECZ400 (400 MHz) spectrometer (JEOL Ltd., Tokyo, Japan) with solvent peaks as references. The electrospray ionization time-of-flight (ESI-TOF) mass spectra were recorded on a LCT Premier XE mass spectrometer (Waters Corporation, Milford, MA, USA).

^1^H–{^13^C–^15^N} Triple-resonance NMR spectra were recorded at 298 K on a Avance 600 spectrometer (600 MHz) equipped with a 5 mm TCI CryoProbe (Bruker BioSpin GmbH, Rheinstetten, Germany), using a previously reported ^1^H–{^13^C–^15^N} pulse sequence for 1D triple-resonance measurements [21]. The parameters used for the detection of ^1^H–^13^C–^15^N in nicotinamide riboside (NR) were transmitter offsets of ^13^C = 100 ppm and ^15^N = 217 ppm, and delay intervals of 1/4*J*_CH_ = 1.41 ms and 1/4*J*_CN_ = 43.9 ms. Those used for the detection of nicotinamide adenine dinucleotide (NAD^+^/NADH) were transmitter offsets of ^13^C = 100 ppm and ^15^N = 100 ppm, and delay intervals of 1/4*J*_CH_ = 1.41 ms and 1/4*J*_CN_ = 17.5 ms. Data processing and analysis were performed using the Topspin 3.1 software (Bruker Biospin GmbH, Rheinstetten, Germany).

### 2.2. Preparation of ^13^C/^15^N-Nicotinamide Riboside

#### 2.2.1. [1-^13^C]-1′-O-Methyl-*α/β*-d-Ribofuranoside 1

A solution of [1-^13^C]-d-ribofuranose (98%, ^13^C) (723 mg, 4.82 mmol) and Dowex^®^ (50 W × 8) cationic exchange resin (Fujifilm Wako, Osaka, Japan) (1.10 g) in methanol (15 mL) was stirred at −4 °C for 24 h. After insoluble solid was removed by filtration through Celite^®^ filter aid (Fujifilm Wako, Osaka, Japan), the filtrate was concentrated *in vacuo* to give compound **1** (*α*/*β*-mixture) (782 mg, quant.) as a colorless oil; ^1^H NMR (CD_3_OD, 500 MHz) *δ*: 4.73 (*d*, *J*_CH_ = 171.8 Hz, 1H), 4.04–4.02 (*m*, 1H), 4.00–3.90 (*m*, 1H), 3.86 (*d*, *J* = 4.6 Hz, 1H), 3.72 (*dd*, *J* = 4.6, 12.0 Hz, 1H), 3.53 (*dd*, *J* = 6.3, 12.0 Hz, 1H), 3.31 (*s*, 3H) for *β*-configuration; ^13^C{^1^H} NMR (D_2_O, 125 MHz) *δ*: 108.5 for labeled C.

#### 2.2.2. [1-^13^C]-1′-O-Methyl-*α/β*-d-Ribofuranoside-2,3,5-Triacetate 2

A solution of compound **1** (692 mg, 4.22 mmol) and acetic anhydride (2.0 mL) in pyridine (10 mL) was stirred at −4 °C for 12 h. The reaction mixture was poured into ice water and extracted with ethyl acetate. The organic layer was dried with MgSO_4_ and concentrated in vacuo. The crude residue was purified by column chromatography (SiO_2,_ ethyl acetate/hexane = 3:1) to give compound **2** (1.06 g, 86%) as a yellow oil; ^1^H NMR (CD_3_OD, 500 MHz) *δ*: 5.30 (*dd*, *J* = 4.6, 5.2 Hz, 1H), 5.19 (*d*, *J* = 4.6 Hz, 1H), 4.92 (*d*, *J*_CH_ = 175.8 Hz, 1H), 4.36 (*dd*, *J* = 1.2, 12.0 Hz, 1H), 4.26–4.25 (*m*, 1H), 4.13 (*dd*, *J* = 5.2, 12.0 Hz 1H), 3.35 (*s*, 3H), 2.05 (*s*, 9H) for *β*-configuration; ^13^C{^1^H} NMR (CD_3_OD, 125 MHz) *δ*: 106.2 for labeled C.

#### 2.2.3. [1-^13^C]-*β*-d-Ribofuranoside-1′,2′,3′,5′-Tetraacetate 3

Compound **3** was prepared as reported previously [26]. Briefly, a solution of compound **2** (1.05 g, 3.63 mmol), acetic anhydride (9.6 mL), and sulfuric acid (0.3 mL) in acetic acid (4.8 mL) was stirred at −4 °C for 8 h. The reaction mixture was poured into ice-cold water and extracted with ethyl acetate. The organic layer was dried with MgSO_4_ and concentrated *in vacuo*. The crude residue was purified by column chromatography (SiO_2_, ethyl acetate/hexane = 4:1) and then recrystallized from 2-propanol to give compound **3** (243 mg, 29%) as a white solid; ^1^H NMR (CD_3_OD, 500 MHz) *δ*: 6.13 (*d*, *J* = 182.7 Hz, 1H), 5.39 (*dd*, *J* = 4.6, 6.0 Hz, 1H), 5.36 (*d*, *J* = 6.0 Hz, 1H), 4.38 (*dd*, *J* = 4.6, 5.2 Hz, 1H), 4.35 (*d*, *J* = 11.5 Hz, 1H), 4.21 (*dd*, *J* = 5.2, 11.5 Hz, 1H), 2.12–2.00 (*m*, 12H); ^13^C{^1^H} NMR (CD_3_OD, 125 MHz,) *δ*: 98.3 for labeled C and 170.9, 170.1, 170.0, 169.3, 79.6, 74.5, 74.1, 70.6, 63.3, 19.7, 19.4, 19.1 for non-labeled C.

#### 2.2.4. [1-^13^C, ^15^N]-*β*-Nicotinamide Riboside (^13^C/^15^N-NR)

A solution of ^15^N-nicotinamide (157 mg, 1.28 mmol) and trimethylsilyl chloride (TMSCl) (0.33 mL) in hexamethyldisilazane (HMDS) (4.5 mL) was refluxed for 12 h and concentrated in vacuo. Next, the residue containing trimethylsilyl-protected ^15^N-nicotinamide was dissolved in dichloroethane (15.0 mL). Compound **3** (406 mg, 1.28 mmol) and trimethylsilyl trifluoromethanesulfonate (TMSOTf) (0.26 mL, 1.42 mmol) were then added, and the mixture was stirred for 3 h at 45 °C. The reaction mixture was concentrated *in vacuo*. The residue was dissolved in ammonia (2 M) in methanol (25 mL) to remove the protective acetyl groups. The mixture was stirred at −4 °C for 10 h and concentrated *in vacuo*. The crude residue was purified by reversed-phase column chromatography with water to give ^13^C/^15^N-labeled NR (326 mg, 63%) as a colorless oil; ^1^H NMR (D_2_O, 500 MHz) *δ*: 9.60 (*s*, 1H), 9.27 (*s*, 1H), 8.98 (*d*, *J* = 8.0 Hz, 1H), 8.28 (*dd*, *J* = 11.5, 6.9 Hz, 1H), 6.24 (*d*, *J*_CH_ = 177.6 Hz, 1H), 4.49 (*d*, *J* = 14.9 Hz, 2H), 4.37–4.35 (*m*, 1H), 4.05 (*d*, *J* = 13.2 Hz, 1H), 3.90 (*dd*, *J* = 12.9, 3.7 Hz, 1H); ^13^C{^1^H} NMR (CD_3_OD, 100 MHz) *δ*: 102.5 (*d*, *J*_CN_ = 5.8 Hz) for labeled C; ^15^N{^1^H} NMR (D_2_O, 40 MHz) *δ*: 215.3 (*d*, *J*_CN_ = 6.2 Hz) for labeled N; HRMS (ESI-TOF): *m/z* calcd. for C_10_^13^CH_15_N_1_^15^NO_5_ [M + H]^+^ = 257.0979, found = 257.0985.

The 1D NMR spectra (^1^H, ^13^C, and ^15^N) and 2D HSQC spectra for ^13^C/^15^N-labeled NR are shown in the Supplementary Materials (Figures S1–S4).

### 2.3. Reduction of ^13^C/^15^N-Nicotinamide Riboside with Na_2_S_2_O_4_

A solution of ^13^C/^15^N-NR (30 mg, 73.8 μmol) and Na_2_S_2_O_4_ (17 mg, 83.6 μmol) and NaHCO_3_ (27 mg, 209 μmol) in D_2_O was stirred for 2 h at room temperature. The reaction mixture was rapidly subjected to NMR analysis. Formation of the reduced NR species (^13^C/^15^N-NRH) was confirmed by ^1^H NMR, ^13^C NMR, and high-resolution ESI-TOF MS; ^1^H-NMR (D_2_O, 600 MHz) *δ*: 7.09 (*s*, 1H), 6.04 (*s*, 1H), 4.82 (*d*, *J*_CH_ = 141.1 Hz, 1H), 4.13 (*s*, 1H), 3.80–3.52 (*m*, 6H), 2.99 (*s*, 1H); ^13^C NMR (125 MHz, D_2_O) *δ*: 94.9 (*d*, *J*_CN_ = 14.4 Hz) for labeled C; HRMS (ESI-TOF): *m*/*z* calcd. for C_10_^13^CH_16_N_1_^15^NO_5_ [M + H]^+^ = 258.1093, found = 258.1112.

### 2.4. Ethics of Animal Experiments

All animal experiments using ICR mice (7 weeks of age, female) obtained from Japan SLC, Inc. were approved by the animal experiment committee and conducted in accordance with the Institutional Guidelines of Tokushima University.

### 2.5. Triple-Resonance NMR Analysis of Probe-Derived NAD^+^ in Mouse Liver Lysate After Administration of ^13^C/^15^N-labeled NR

Female ICR mice weighing ~35 g were injected intraperitoneally with an aqueous saline solution of ^13^C/^15^N-NR (1.83 mmol/kg body weight) and sacrificed 2 h later by cervical dislocation. The whole liver (1.56 g) was excised and homogenized in a cold lysis solution (methanol/chloroform = 1:2) using a TissueLyser LT (QIAGEN K.K., Tokyo, Japan) at 4 °C for 3 min. The homogenate was then centrifuged at 3000 rpm at 4 °C for 3 min and washed with the lysis solution. The extract corrected was lyophilized to dryness, dissolved in D_2_O (500 μL) in a nitrogen-filled glove box, and subjected to NMR analysis (256 scans). The present experiment was performed as an exploratory proof-of-concept analysis using a liver extract prepared from an individual mouse.

### 2.6. Triple-Resonance NMR Analysis of Probe-Derived NAD^+^ and NADH Signals in an Ethanol Treated Mouse Model

An ethanol-treated mouse model was prepared as previously reported [27]. Briefly, an aqueous saline solution containing 50% ethanol (5 g/kg body weight) was administered orally to female ICR mice weighing approximately 35 g every 12 h for a total of two doses. The mice were used for the experiment 10 h after the final ethanol administration.

The model mice were injected intraperitoneally with an aqueous saline solution of ^13^C/^15^N-NR (1.83 mmol/kg) and sacrificed by cervical dislocation 2 h later. The whole liver (1.23 g) was harvested and homogenized in the same cold lysis solution described above to prepare the extract. The combined extract was lyophilized to dryness, dissolved in D_2_O (500 μL) in a nitrogen-filled glove box, and subjected to NMR analysis (1024 scans). The present comparison was performed as an exploratory proof-of-concept analysis using liver extracts prepared from individual control and ethanol-treated mice.

## 3. Results

### 3.1. Synthesis and Triple-Resonance NMR Analysis of ^13^C/^15^N-Labeled Nicotinamide Riboside

**Scheme 1:**
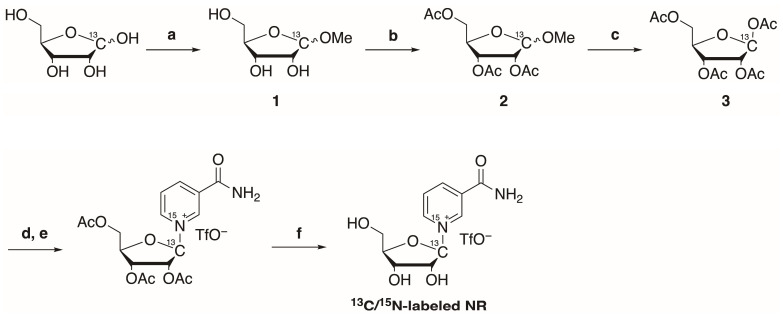
Synthesis of ^13^C/^15^N-labeled nicotinamide riboside (^13^C/^15^N-NR). Reagents, solvent, yield: (a) Dowex ion exchange resin (H^+^), MeOH, quant.; (b) Ac_2_O, pyridine, AcOH, 86%; (c) Ac_2_O, H_2_SO_4_, AcOH, 29%; (d) ^15^N-nicotinamide, HMDS, TMSCl; (e) TMSOTf, 1,2-dichloroethane; (f) 2 M NH_3_/MeOH, 63% (for three steps).

As shown in Scheme 1, ^13^C/^15^N-NR was synthesized from ^13^C-labeled d-ribofuranose and ^15^N-labeled nicotinamide as the respective isotope sources. The starting material, [1-^13^C]-d-ribofuranose, was converted into the 1′-*O*-methylated derivative **1** in quantitative yield by treatment with a cation-exchange resin in methanol. Compound **1** was then protected to afford the 2′,3′,5′-*O*-triacetylated derivative **2**, which was subsequently transformed into 1′,2′,3′,5′-*O*-acetyl-*β*-d-ribofuranose **3** using acetic anhydride containing a catalytic amount of sulfuric acid. Fully acetylated *β*-ribofuranose **3** was coupled with TMS-protected ^15^N-nicotinamide in the presence of TMSOTf, and subsequent deprotection with aqueous NH_3_ afforded the ^13^C/^15^N-NR probe exclusively in the *β*-anomeric form.

**Figure 2:**
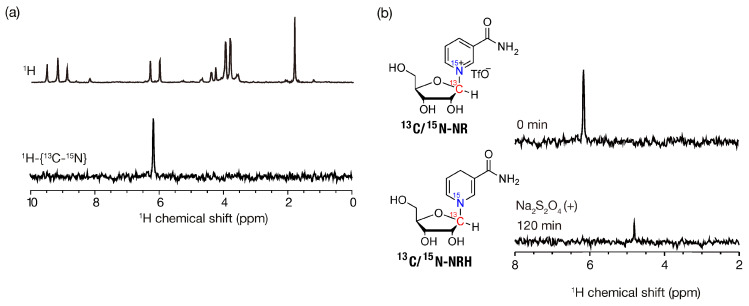
(**a**) ^1^H (**top**) and ^1^H–{^13^C–^15^N} NMR (**bottom**) spectra of ^13^C/^15^N-NR (5 mM) in D_2_O using NR-optimized triple-resonance pulse conditions. (**b**) Time course of the change in ^1^H–{^13^C–^15^N} NMR spectra of ^13^C/^15^N-NR in D_2_O containing Na_2_S_2_O_4_, incubated at room temperature for 0 (**top**) or 120 min (**bottom**), using NR-optimized pulse conditions. The NMR spectra were obtained with a 600 MHz NMR spectrometer equipped with a high-sensitivity cryogenic probe after 16 scans (~1 min).

Figure 2a shows the ^1^H (top) and ^1^H–{^13^C–^15^N} triple-resonance (bottom) NMR spectra of ^13^C/^15^N-labeled NR in D_2_O. ^1^H-NMR spectrum of ^13^C/^15^N-labeled NR showed a strongly coupled split peak at 6.18 ppm, which was assigned as ^13^CH1′ peak (*J*_CH_ = 177.5 Hz) (Figure 2a, top). We then acquired the triple-resonance NMR spectrum of the ^13^C/^15^N-labeled NR using a previously reported pulse sequence [21] designed to detect a ^1^H nucleus bound to ^13^C1′, which is in turn coupled to the pyridyl ^15^N1 nucleus of NR (NR-optimized triple-resonance NMR parameters; ^13^C = 100 ppm and ^15^N = 217 ppm, and 1/4*J*_CH_ = 1.41 ms and 1/4*J*_CN_ = 43.9 ms). Under these conditions, the triple-resonance ^1^H–^13^C–^15^N spectrum of ^13^C/^15^N-labeled NR in D_2_O displayed a single peak at 6.18 ppm, attributable to the proton attached at the ribose C1′ (Figure 2a, bottom), which was confirmed independently using 2D ^1^H–^13^C HSQC analysis (Figure S4).

Next, we examined the change in the chemical shift of the methine proton of NR under reducing conditions. Excess Na_2_S_2_O_4_ was added to the solution containing ^13^C/^15^N-NR, and formation of the reduced NR species (^13^C/^15^N-NRH) was confirmed with ^1^H NMR, ^13^C NMR, and high-resolution ESI-TOF MS. The triple-resonance spectrum of ^13^C/^15^N-NRH exhibited a single signal at 4.81 ppm (Figure 2b, bottom), indicating that reduction of the pyridyl ring induces a substantial chemical shift change in the C1′ methine proton within the ^1^H–^13^C1′–^15^N1 spin system. The weaker NRH signal is likely attributable to the use of NR-optimized pulse parameters (^13^C = 100 ppm, ^15^N = 217 ppm, *J*_C–H_ = 177.5 Hz, *J*_C–N_ = 5.7 Hz), as reduction of the pyridyl ring alters the ^15^N chemical shift and ^1^H–^13^C–^15^N coupling conditions. This result indicates that appropriate pulse-parameter optimization is important for the simultaneous detection of oxidized and reduced species in the ^1^H–^13^C–^15^N spin system. This pronounced contrast clearly demonstrates that labeled NR and its reduced counterpart function effectively as probes for ^1^H–^13^C–^15^N triple-resonance NMR analysis and can be selectively detected during the NR/NRH redox process.

**Figure 3:**
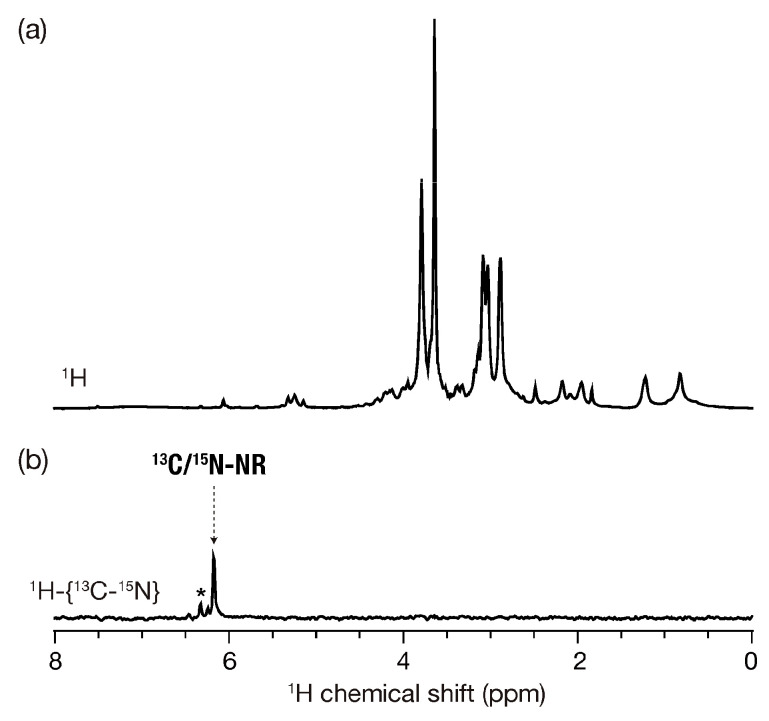
^1^H (**a**) and NR-optimized ^1^H–{^13^C–^15^N} (**b**) NMR spectra of mouse liver lysate (10% *v*/*v*) containing ^13^C/^15^N-NR (4 mM). Single- and triple-resonance spectra were obtained after 16 and 256 scans, respectively. The asterisk denotes noise derived from the lysate buffer solutions.

We also performed NMR measurements of the NR probe (4 mM) in the presence of inactivated mouse liver lysate (10% *v*/*v*) to evaluate its performance under biologically complex conditions. In the conventional ^1^H NMR spectrum, selective observation of the NR probe was not feasible because all ^1^H-containing molecules in the lysate contributed to the detected signals (Figure 3a). In contrast, the ^1^H–{^13^C–^15^N} triple-resonance spectrum displayed a single peak at 6.18 ppm (Figure 3b), thereby confirming the utility of the triple-resonance NMR method for the selective detection of the labeled probe.

### 3.2. Triple-Resonance NMR Analysis of ^13^C/^15^N-Labeled Nicotinamide Riboside in the Mouse Liver

**Figure 4:**
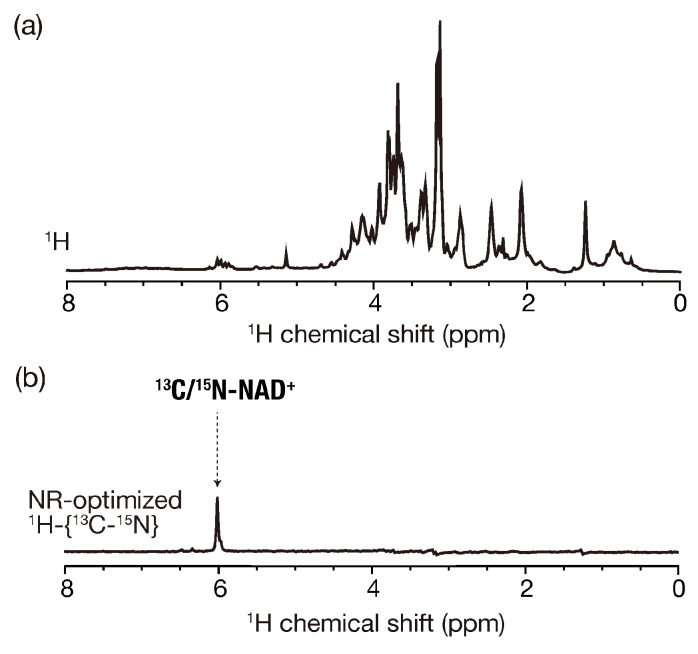
Conversion of a ^13^C/^15^N-NR probe to NAD^+^ in the mouse liver. NMR spectra of liver tissue extracts from a mouse. Conventional ^1^H (**a**) and NR-optimized ^1^H–{^13^C–^15^N} triple-resonance (**b**) NMR spectra of liver extracts from mice administered with ^13^C/^15^N-NR (1.83 mmol/kg).

Next, we performed the triple-resonance NMR measurements to investigate the conversion of ^13^C/^15^N-NR to labeled NAD^+^ in mice. ^13^C/^15^N-NR (1.83 mmol/kg body weight) was administered intraperitoneally to a mouse (~34 g). After 2 h, the liver was collected, lysed, and re-dissolved in D_2_O, and then subjected to NMR analysis. As shown in Figure 4a, the conventional ^1^H spectrum did not permit the reliable detection of either the administered probe (NR) or its metabolic products (NAD^+^/NADH) because of the numerous signals originating from endogenous hepatic components. In marked contrast, the 1D ^1^H–{^13^C–^15^N} triple-resonance spectrum acquired under NR-optimized pulse conditions revealed a distinct single peak at 6.01 ppm (Figure 4b), which was assigned to the proton signal derived from the ^1^H–^13^C1′–^15^N1 arrangement of labeled NAD^+^, as independently confirmed by two-dimensional ^1^H–^13^C HSQC analysis. Under the NR-optimized pulse conditions (^13^C = 100 ppm, ^15^N = 217 ppm, *J*_C–H_ = 177.5 Hz, *J*_C–N_ = 5.7 Hz), no signal corresponding to labeled NADH was observed. In addition, previous studies have shown that enzymatic conversion of NR to NAD^+^ in the liver proceeds rapidly [28]. Collectively, these results indicate that ^13^C/^15^N-NR is converted to labeled NAD^+^ in mouse liver tissue, and that the product can be monitored specifically by triple-resonance NMR.

### 3.3. Triple-Resonance NMR Monitoring of Probe-Derived NAD^+^ and NADH Signals in the Mouse Liver

**Figure 5:**
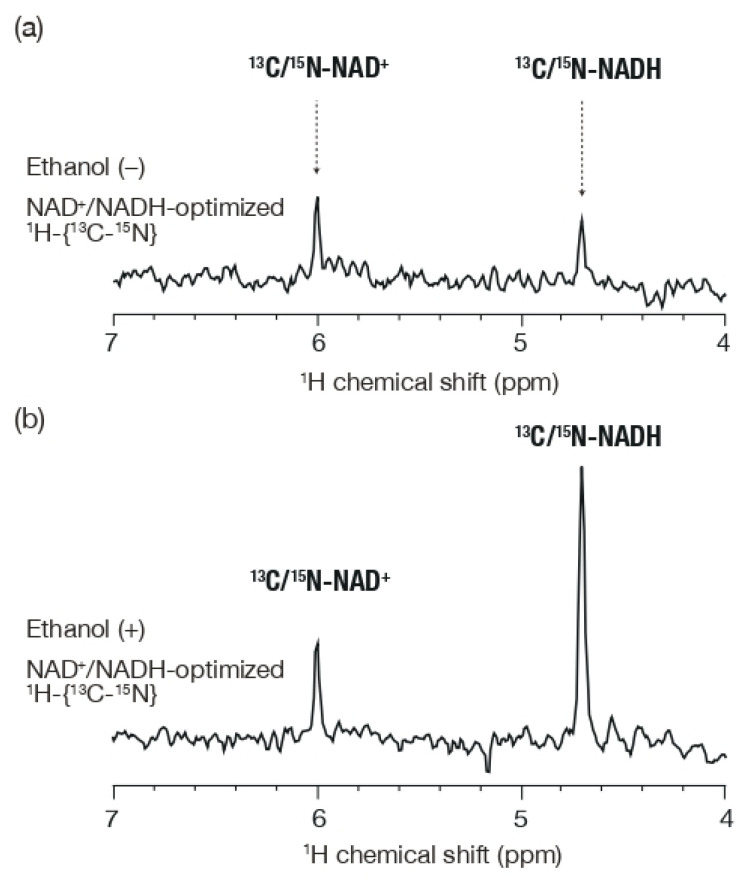
NAD^+^/NADH-optimized ^1^H–{^13^C–^15^N} NMR spectra of liver extracts from mice administered (**a**) ^13^C/^15^N-NR (1.83 mmol/kg) and (**b**) 50% ethanol (5 g/kg) and ^13^C/^15^N-NR (1.83 mmol kg^−1^). NMR spectra were acquired after 1024 scans under NAD^+^/NADH-optimized pulse conditions. Signal-to-noise ratios were calculated for the labeled NAD^+^ and NADH signals. Representative spectra obtained from individual mice under control and ethanol-treated conditions.

We subsequently examined changes in probe-derived NAD^+^- and NADH-related signals in an ethanol-treated mouse model. In the liver, ethanol is metabolized through reactions catalyzed by alcohol dehydrogenase and aldehyde dehydrogenase, both of which convert NAD^+^ to NADH [29]. Model mice (~36 g) administered 50% ethanol (5 g/kg) orally were intraperitoneally injected ^13^C/^15^N-NR (1.83 mmol/kg). After 2 h, the liver was collected, and after work up, the triple-resonance spectra were obtained under NAD^+^/NADH-optimized pulse conditions. Because the chemical shift and scalar coupling of the ^15^N nucleus in the pyridyl ring of NADH differ substantially from those of NAD^+^ [24], triple-resonance NMR measurements were performed using pulse parameters optimized for the simultaneous detection of NAD^+^ and NADH in the lysate (^13^C = 100 ppm, ^15^N = 100 ppm, *J*_C–H_ = 177.5 Hz, *J*_C–N_ = 14.3 Hz). Under these conditions, the triple-resonance NMR spectra exhibited two signals: at 6.01 ppm for labeled NAD^+^ and 4.71 ppm for labeled NADH (Figure 5). The relative signal-to-noise (S/N) ratio between the labeled NAD^+^ and NADH decreased approximately fivefold in the ethanol-treated group (Figure 5b) relative to the control group (Figure 5a), with apparent values of 1.66 and 0.33 for the control and ethanol-treated groups, respectively, after normalization to liver tissue weight. This apparent change in the relative signal ratio was nearly consistent with values previously reported for mice using an enzymatic cycling assay [30].

## 4. Discussion

A major advantage of the NMR-based approach is its potential applicability to magnetic resonance spectroscopy (MRS) for in vivo observation of biomolecules. In the present study, we designed a stable isotope-labeled NAD^+^ precursor, the ^13^C/^15^N-NR probe, which undergoes enzymatic conversion to NAD^+^ and NADH in the mouse liver. Using this probe, we selectively detected probe-derived NAD^+^ and NADH signals in mouse liver lysates by ^1^H–{^13^C–^15^N} triple-resonance NMR measurements. Our findings provide a technical foundation for the selective and simultaneous detection of NAD^+^- and NADH-related signals by exploiting their pronounced chemical shift differences and suggest the future feasibility of in vivo MRS applications [31].

The present study should be regarded as a methodological proof-of-concept, and the results should be interpreted within this scope. Because the current method selectively detects probe-derived NAD^+^ and NADH generated from exogenously administered ^13^C/^15^N-NR, it is suited to tracking probe-traceable redox-related changes in liver extracts, rather than directly quantifying the total endogenous NAD^+^/NADH pool. In addition, the observed signal changes were obtained under the present experimental conditions, including probe administration and exploratory biological sampling, and should therefore be interpreted as probe-derived redox-related signal changes rather than as a fully validated quantitative measure of the endogenous hepatic redox state. These considerations define the current scope of the present study and should be taken into account in future methodological development and validation.

To date, in vivo ^1^H and ^31^P MR spectroscopy for the detection of NAD^+^ and NADH in biological tissues has remained challenging. In ^31^P MR spectroscopy, NAD(H) has been evaluated through the ^31^P NMR signals arising from the phosphate diester moieties of NAD(H). Because the ^31^P signals of NAD(H) overlap extensively with one another and with signals from uridine diphosphate glucose, analysis generally requires spectral deconvolution of the individual NAD^+^ and NADH signals [32,33]. More recently, Mevenkamp et al. developed a ^31^P MRS-sequence that improves NAD^+^ and NADH detection by suppressing the α-ATP signal, which normally overlaps with these resonances [34]. Although ^1^H NMR signals are potentially more sensitive than ^31^P NMR signals, ^1^H-MRS permits the observation of NAD^+^ primarily through the detection of the pyridyl ring protons of NAD^+^. However, in NADH, these protons are shifted upfield, resulting in spectral overlap with ATP and other metabolites [35]. A recent report by Gowda et al. [36,37] used ex vivo ^1^H NMR spectroscopy to detect coenzymes, including NAD^+^ and NADH, in mouse liver extracts. That method can provide information related to NAD(H) redox balance by monitoring adenine-derived ^1^H signals of NAD(H), which appear in the downfield region with relatively limited interference from impurities. Nevertheless, the use of proton signals with closely similar chemical shifts is not well-suited to in vivo ^1^H-MRS measurements [35]. Therefore, further improvements in the selectivity of ^1^H NMR signals obtained by ^1^H-based NMR methods, including multiple-resonance NMR, will be important for future in vivo studies of NAD-related redox dynamics.

Future studies should focus on improving the quantitative evaluation of individual NMR signals, including calibration and signal-response correction, and on extending the applicability of this approach to biologically relevant experimental settings. Such efforts will further establish the utility of probe-based triple-resonance NMR as a selective strategy for monitoring NAD-related redox changes.

## Data Availability

Data is contained within the article or Supplementary Materials.

## Supplementary Materials

The following supporting information can be downloaded at: https://www.mdpi.com/article/10.3390/s26092714/s1, Figure S1: ^1^H NMR spectrum of [1-^13^C, ^15^N]-*β*-nicotinamide riboside (^13^C/^15^N-NR) in D_2_O (500 MHz); Figure S2: ^13^C{^1^H} NMR spectrum of [1-^13^C, ^15^N]-*β*-nicotinamide riboside (^13^C/^15^N-NR) in CD_3_OD (100 MHz); Figure S3: ^15^N{^1^H} NMR spectrum of [1-^13^C, ^15^N]-*β*-nicotinamide riboside (^13^C/^15^N-NR) in D_2_O (40 MHz); Figure S4: 2D ^1^H-^13^C HSQC spectrum of [1-^13^C, ^15^N]-*β*-nicotinamide riboside (^13^C/^15^N-NR) in D_2_O (600 MHz).

## References

[1] Cantó C., Menzies K.J., Auwerx J. (2015). NAD^+^ metabolism and the control of energy homeostasis: A balancing act between mitochondria and the nucleus. Cell Metab..

[2] Hou Y., Lautrup S., Cordonnier S., Wang Y., Croteau D.L., Zavala E., Zhang Y., Moritoh K., O’Connell J.F., Baptiste B.A. (2018). NAD^+^ supplementation normalizes key Alzheimer’s features and DNA damage responses in a new AD mouse model with introduced DNA repair deficiency. Proc. Natl. Acad. Sci. USA.

[3] Karamanlidis G., Lee C.F., Garcia-Menendez L., Kolwicz S.C., Suthammarak W., Gong G., Sedensky M.M., Morgan P.G., Wang W., Tian R. (2013). Mitochondrial complex I deficiency increases protein acetylation and accelerates heart failure. Cell Metab..

[4] Sun L.J., Li S.C., Zhao Y.H., Yu J.W., Kang P., Yan B.Z. (2013). Silent information regulator 1 inhibition induces lipid metabolism disorders of hepatocytes and enhances hepatitis C virus replication. Hepatol. Res..

[5] Migaud M.E., Zieglar M., Baur J.A. (2024). Regulation and challenges in NAD^+^ metabolism. Nat. Rev. Mol. Cell Biol..

[6] Lu W., Wang L., Chen L., Hui S., Rabinowitz J.D. (2018). Extraction and quantitation of nicotinamide adenine dinucleotide redox cofactors. Antioxid. Redox Signal..

[7] Qian R.C., Zhao L.J., Lv J., Hua X., Long Y.T. (2018). Reversible redox inter-conversion of biologically active NAD^+^/NADH derivatives bound to a gold electrode: ToF-SIMS evidence. Chem. Commun..

[8] Zhu C.T., Rand D.M. (2012). A hydrazine coupled cycling assay validates the decrease in redox ratio under starvation in *Drosophila*. PLoS ONE.

[9] Umemura K., Kimura H. (2005). Determination of oxidized and reduced nicotinamide adenine dinucleotide in cell monolayers using a single extraction procedure and a spectrophotometric assay. Anal. Biochem..

[10] Zhao Y., Hu Q., Cheng F., Su N., Wang A., Zou Y., Hu H., Chen X., Zhou H.M., Huang X. (2015). SoNar, a highly responsive NAD+/NADH sensor, allows high-throughput metabolic screening of anti-tumor agents. Cell Metab..

[11] Kondo T., Kimura Y., Yamada H., Aoyama Y. (2017). Polymeric ^1^H MRI Probes for Visualizing Tumor In Vivo. Chem. Rec..

[12] Alwahsh M., Nimer R.M., Dahabiyeh L.A., Hamadeneh L., Hasan A., Rahaf A., Hegenroser R. (2024). NMR-based metabolomics identification of potential serum biomarkers of disease progression in patients with multiple sclerosis. Sci. Rep..

[13] Peters J.P., Brahms A., Janicaud V., Anikeeva M., Peschke E., Ellermann F., Ferrari A., Hellmold D., Held-Feindt J., Kim N.-M. (2023). Nitrogen-15 dynamic nuclear polarization of nicotinamide derivatives in biocompatible solution. Sci. Adv..

[14] Yang H., Staveness D., Ryckbosch S.M., Axtman A.D., Loy B.A., Barns A.B., Pande V.S., Schaefer J., Wender P.A., Cegleski L. (2018). REDOR NMR reveals multiple conformers for a protein kinase C ligand in a membrane environment. ACS Cent. Sci..

[15] Cegleski L. (2013). REDOR NMR for drug discovery. Bioorg. Med. Chem. Lett..

[16] Matsuoka S., Inoue S. (2009). Application of REDOR NMR in natural product chemistry. Chem. Commun..

[17] Kay L.E., Ikura M., Tschudin R., Bax A. (1990). Three-dimensional triple-resonance NMR spectroscopy of isotopically enriched proteins. J. Magn. Reson..

[18] Fan T.W.-M., Lane A.N. (2008). Structure-based profiling of metabolites and isotopomers by NMR. Prog. Nucl. Magn. Reson. Spectrosc..

[19] Chikayama E., Suto M., Nishihara T., Shinozaki K., Hirayama T., Kikuchi J. (2008). Systematic NMR analysis of stable isotope labeled metabolite mixtures in plant and animal systems: Coarse grained views of metabolic pathways. PLoS ONE.

[20] Hutton W.C., Likos J.J., Gard J.K., Garbow J.R. (1998). Triple resonance isotope-edited (TRIED): A powerful new NMR technique for studying metabolism. J. Label. Compd. Radiopharm..

[21] Yamada H., Mizusawa K., Igarashi R., Tochio H., Shirakawa M., Tabata Y., Kimura Y., Kondo T., Aoyama Y., Sando S. (2012). Substrate/product-targeted NMR monitoring of pyrimidine catabolism and its inhibition by a clinical drug. ACS Chem. Biol..

[22] Yamada H., Kameda T., Kimura Y., Imai H., Matsuda T., Sando S., Toshimitsu A., Aoyama Y., Kondo T. (2016). ^13^C/^15^N-Enriched l-Dopa as a triple-resonance NMR probe to monitor neurotransmitter dopamine in the brain and liver extracts of mice. ChemistryOpen.

[23] Ratajczak J., Joffraud M., Trammell S.A.J., Ras R., Canela N., Boutant M., Kulkarni S.S., Rodrigues M., Redpath P., Migaud M.E. (2016). NRK1 controls nicotinamide mononucleotide and nicotinamide riboside metabolism in mammalian cells. Nat. Commun..

[24] Oppenheimer N.J., Davidson R.M. (1980). ^15^N nuclear magnetic resonance studies of [1-^15^N]nicotinamide adenine dinucleotides. Org. Magn. Reson..

[25] Shchepin R.V., Barskiy D.A., Mikhaylov D.M., Chekmenev E.Y. (2016). Efficient synthesis of nicotinamide-1-^15^N for ultrafast NMR hyperpolarization using parahydrogen. Bioconjug. Chem..

[26] Patching S.G., Baldwin S.A., Baldwin A.D., Young J.D., Gallagher M.P., Henderson P.J.F., Herbert R.B. (2005). The nucleoside transport proteins, NupC and NupG, from *Escherichia coli*: Specific structural motifs necessary for the binding of ligands. Org. Biomol. Chem..

[27] Khanal T., Choi J.H., Hwang Y.P., Chung Y.C., Jeong H.G. (2009). Saponins isolated from the root of *Platycodon grandiflorum* protect against acute ethanol-induced hepatotoxicity in mice. Food Chem. Toxicol..

[28] Khan J.A., Xiang S., Tong L. (2007). Crystal structure of human nicotinamide riboside kinase. Structure.

[29] Yu J.H., Song S.J., Kim A., Choi Y., Seok J.W., Kim H.J., Lee Y.J., Lee K.S., Kim J.W. (2016). Suppression of PPARγ-mediated monoacylglycerol *O*-acyltransferase 1 expression ameliorates alcoholic hepatic steatosis. Sci. Rep..

[30] Marmier S., Dentin R., Daujat-Chavanieu M., Guillou H., Bertrand-Michel J., Gerbal-Chaloin S., Girard J., Lotersztajn S., Postic C. (2015). Novel role for carbohydrate responsive element binding protein in the control of ethanol metabolism and susceptibility to binge drinking. Hepatology.

[31] Yamada H., Hasegawa Y., Imai H., Takayama Y., Sugihara F., Matsuda T., Tochio H., Shirakawa M., Sando S., Kimura Y. (2015). Magnetic resonance imaging of tumor with a self-traceable phosphorylcholine polymer. J. Am. Chem. Soc..

[32] de Graaf R.A., De Feyter H.M., Brown P.B., Nixon T.W., Rothman D.L., Behar K.L. (2017). Detection of cerebral NAD^+^ in humans at 7T. Magn. Reson. Med..

[33] Lu M., Zhu X.H., Chen W. (2016). In vivo ^31^P MRS assessment of intracellular NAD metabolites and NAD^+^/NADH redox state in human brain at 4 T. NMR Biomed..

[34] Mevenkamp J., Bruls Y.M.H., Mancilla R., Grevendonk L., Wildberger J.E., Brouwers K., Hesselink M.K.C., Schrauwen P., Hoeks J., Houtkooper R.H. (2024). Development of a ^31^P magnetic resonance spectroscopy technique to quantify NADH and NAD^+^ at 3 T. Nat. Commun..

[35] de Graaf R.A., Behar K.L. (2014). Detection of cerebral NAD^+^ by in vivo ^1^H NMR spectroscopy. NMR Biomed..

[36] Nagana Gowda G.A., Abell L., Lee C.F., Tian R., Raftery D. (2016). Anomalous dynamics of labile metabolites in cold human blood detected using ^1^H NMR spectroscopy. Anal. Chem..

[37] Nagana Gowda G.A., Abell L., Tian R., Raftery D. (2023). Whole body distribution of labile coenzymes and antioxidants in a mouse model as visualized using ^1^H NMR Spectroscopy. Anal. Chem..

