# Supplementary material for: A Triple-Resonance NMR Strategy for the Selective Detection of NAD+ and NADH Derived from a 13C/15N-Nicotinamide Riboside Probe in the Liver Extracts of Mice
Source: Sensors (Basel). 2026 Apr 28;26(9):2714. doi: 10.3390/s26092714 (PMC13165850; doi:10.3390/s26092714)
Supplement: Supplementary file 1 [file sensors-26-02714-s001.zip › sensors-4251987-supplementary.pdf]

Supporting Information

for

A Triple-Resonance NMR Strategy for the Selective Detection of NAD^+ and NADH Derived from a $^{13}C/^{15}N$-Nicotinamide Riboside Probe in the Liver Extracts of Mice

Hiroki Shimada [1,†], Yusei Shinohara [1,†], Yoshihiro Uto [1], and Hisatsugu Yamada [1,*]

1 Graduate School of Technology, Industrial and Social Sciences, Tokushima University, 2-1 Minamijyosanjima-cho, Tokushima 770-8506, Japan; hirokishimada10.19@gmail.com (H.S.); shinohara.yuusei@tokushima-u.ac.jp (Y.S.); uto.yoshihiro@tokushima-u.ac.jp (Y.U.)

* Correspondence: yamada.hisatsugu@tokushima-u.ac.jp; Tel.: +81-88-656-7522, FAX: +81-88-656-7522

† These authors contributed equally to this work.

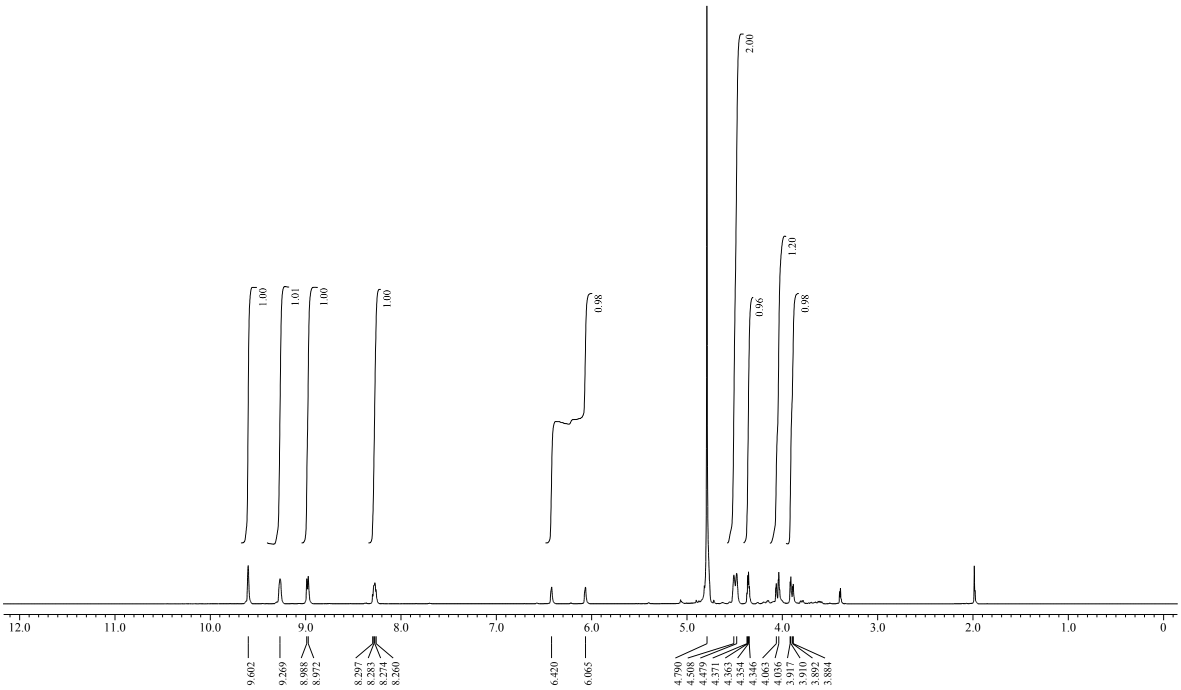

Figure S1. ^{1}H NMR spectrum of [1-^{13}C, ^{15}N]-*β*-nicotinamide riboside (^{13}C/^{15}N-NR) in D_2O (500 MHz).

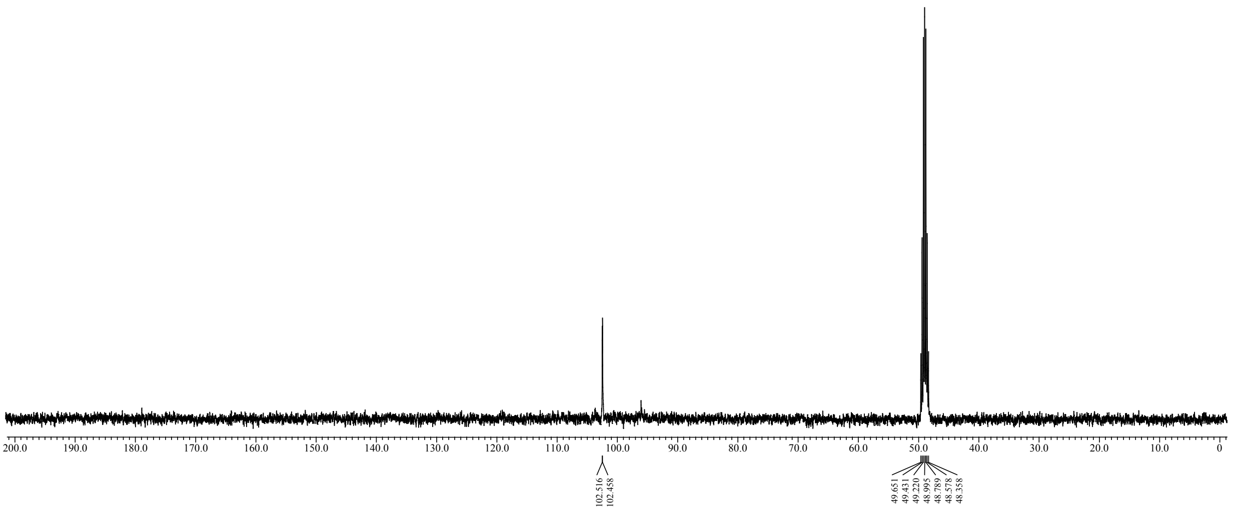

Figure S2. $^{13}C\{^{1}H\}$ NMR spectrum of [1-^{13}C, ^{15}N]-*β*-nicotinamide riboside ($^{13}C/^{15}N$-NR) in CD_3OD (100 MHz).

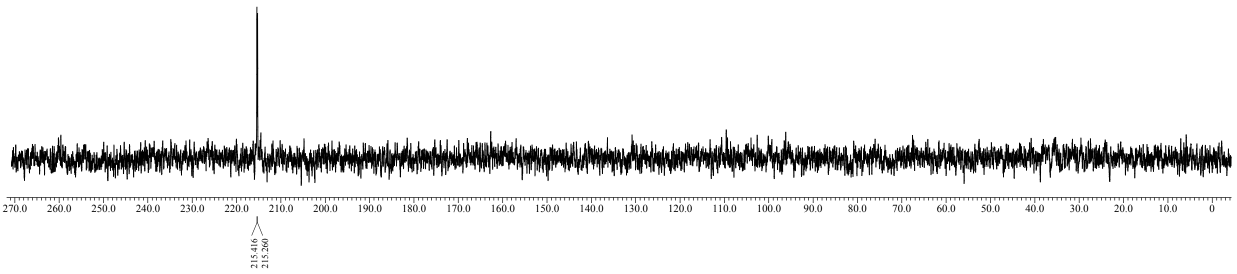

Figure S3. $^{15}N\{^{1}H\}$ NMR spectrum of [1-^{13}C, ^{15}N]-*β*-nicotinamide riboside ($^{13}C/^{15}N$-NR) in D_2O (40 MHz).

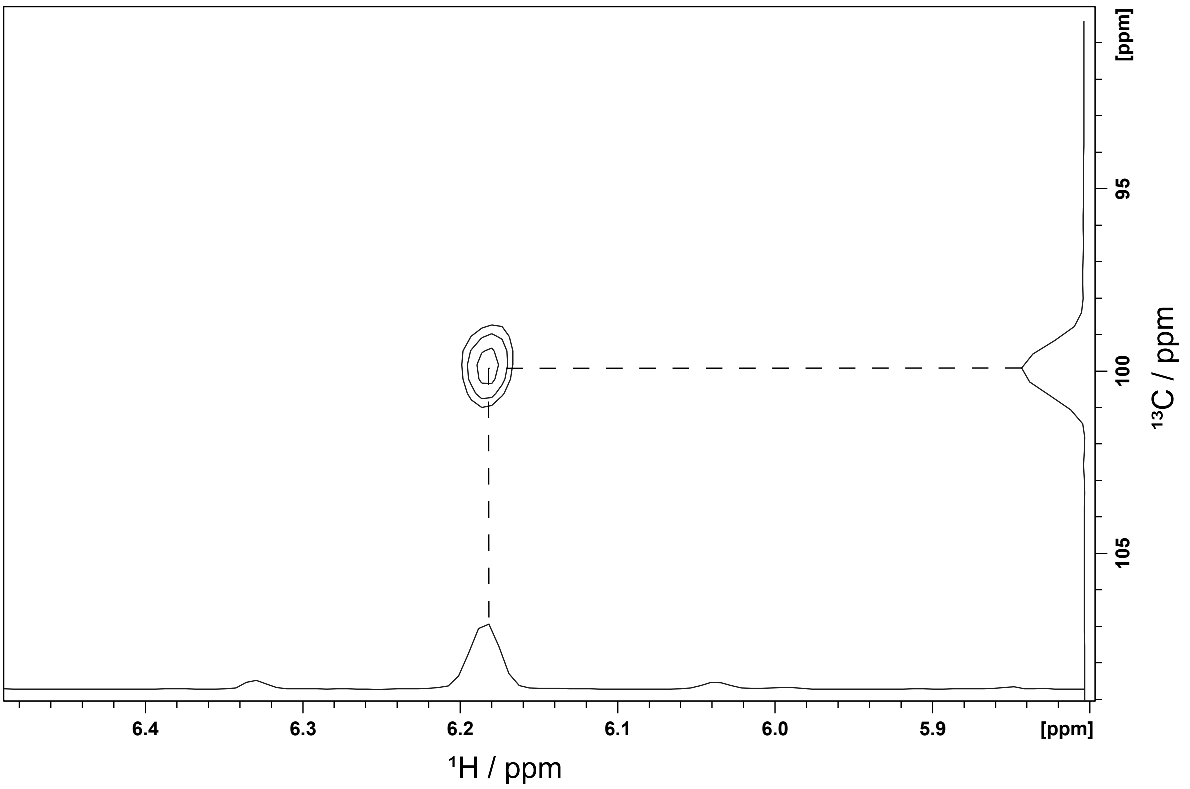

Figure S4. 2D ^1H-^{13}C HSQC spectrum of [1-^{13}C, ^{15}N]-*β*-nicotinamide riboside (^{13}C/^{15}N-NR) in D_2O (600 MHz).
